# Multidisciplinary Team-Based Approach for Comprehensive Preoperative Pulmonary Rehabilitation Including Intensive Nutritional Support for Lung Cancer Patients

**DOI:** 10.1371/journal.pone.0059566

**Published:** 2013-03-15

**Authors:** Hiroaki Harada, Yoshinori Yamashita, Keizo Misumi, Norifumi Tsubokawa, Junichi Nakao, Junko Matsutani, Miyako Yamasaki, Tomomi Ohkawachi, Kiyomi Taniyama

**Affiliations:** 1 Department of Respiratory Surgery, National Hospital Organization Kure Medical Center and Chugoku Cancer Center, Kure, Japan; 2 Institute for Clinical Research, National Hospital Organization Kure Medical Center and Chugoku Cancer Center, Kure, Japan; 3 Department of Surgical Oncology, Research Institute for Radiation Biology and Medicine, Graduate School of Biomedical Sciences, Hiroshima University, Hiroshima, Japan; 4 Department of Rehabilitation, National Hospital Organization Kure Medical Center and Chugoku Cancer Center, Kure, Japan; 5 Department of Nutrition, National Hospital Organization Kure Medical Center and Chugoku Cancer Center, Kure, Japan; The Ohio State Unversity, United States of America

## Abstract

**Background:**

To decrease the risk of postoperative complication, improving general and pulmonary conditioning preoperatively should be considered essential for patients scheduled to undergo lung surgery.

**Objective:**

The aim of this study is to develop a short-term beneficial program of preoperative pulmonary rehabilitation for lung cancer patients.

**Methods:**

From June 2009, comprehensive preoperative pulmonary rehabilitation (CHPR) including intensive nutritional support was performed prospectively using a multidisciplinary team-based approach. Postoperative complication rate and the transitions of pulmonary function in CHPR were compared with historical data of conventional preoperative pulmonary rehabilitation (CVPR) conducted since June 2006. The study population was limited to patients who underwent standard lobectomy.

**Results:**

Postoperative complication rate in the CVPR (n = 29) and CHPR (n = 21) were 48.3% and 28.6% (p = 0.2428), respectively. Those in patients with Charlson Comorbidity Index scores ≥2 were 68.8% (n = 16) and 27.3% (n = 11), respectively (p = 0.0341) and those in patients with preoperative risk score in Estimation of Physiologic Ability and Surgical Stress scores >0.3 were 57.9% (n = 19) and 21.4% (n = 14), respectively (p = 0.0362). Vital capacities of pre- and post intervention before surgery in the CHPR group were 2.63±0.65 L and 2.75±0.63 L (p = 0.0043), respectively; however, their transition in the CVPR group was not statistically significant (p = 0.6815). Forced expiratory volumes in one second of pre- and post intervention before surgery in the CHPR group were 1.73±0.46 L and 1.87±0.46 L (p = 0.0012), respectively; however, their transition in the CVPR group was not statistically significant (p = 0.6424).

**Conclusions:**

CHPR appeared to be a beneficial and effective short-term preoperative rehabilitation protocol, especially in patients with poor preoperative conditions.

## Introduction

Notwithstanding advances in management, complete anatomical resection remains the most effective treatment in patients with early stage lung cancer. According to various guidelines, acceptable mortality following resection varies between 4% and 7% for lobectomy and between 8% and 14% for pneumonectomy [1], [2], [3]. Several investigators have reported increased postoperative mortality ranging up to 11.8% for lobectomy and 16%–20% for pneumonectomy in elderly patients with poor risk compared with those who have adequate pulmonary function [4], [5]. To decrease morbidity and postoperative complication rate, improving or maintaining general conditioning and pulmonary function preoperatively have been considered essential for patients scheduled to undergo lung surgery. Pulmonary rehabilitation has been demonstrated by many investigators to be a beneficial intervention; however, the duration of a standard program was generally 6–12 weeks [6], [7]. Because it is necessary for patients with malignant disease to undergo surgery without delay, effective short-term preoperative pulmonary rehabilitation programs should be adopted.

Branched-chain amino acids (BCAAs) are among the nine essential amino acids for humans, accounting for 35% of the essential amino acids in muscle proteins and 40% of the preformed amino acids required by mammals [8]. BCAAs serve as essential substrates and important regulators in the synthesis of body proteins, especially in body muscles [9]. Several studies have demonstrated that glutamine supplementation, which is produced by BCAAs, improves clinical outcomes and function under some pathological conditions [10], [11]. Recent literature showed that supplementation of BCAA could be beneficial in improving whole-body protein metabolism in COPD patients [12], and that preoperative nutritional status could be an important predictor of morbidity and mortality in patients undergoing surgery for malignant disease [13]–[15]. However, the effect of BCAA supplementation in a short-term preoperative pulmonary rehabilitation program for lung cancer patients has not yet been clarified. Furthermore, it is usually difficult to achieve adequate nutritional intake in elderly patients with pulmonary disorders. For maintenance or improvement of appetite, herbal medications (such as Hochuekkito^TM^; Tsumura Co., Tokyo, Japan) have been recognized as beneficial supplements, and the effects of such medications on COPD have also been evaluated in terms of quality of life [16].

Although several previous studies demonstrated that well-planned short-term preoperative physiotherapy decreased the incidence of postoperative pulmonary complications, we hypothesized that pulmonary rehabilitation with intensive nutritional support could be a beneficial tool in improving the general conditions of lung cancer patients undergoing surgery.

To establish a more enforceable and beneficial program for outpatients, we developed a comprehensive pulmonary rehabilitation (CHPR) protocol, which consists of physical exercise and intensive nutritional support with BCAAs and herbal medication, performed using multidisciplinary team approach. In this study, we analyzed the clinical effect of the CHPR protocol on the outcome of lung cancer patients who underwent standard lobectomy after pulmonary rehabilitation in our hospital.

## Methods

### Study population

**Figure 1:**
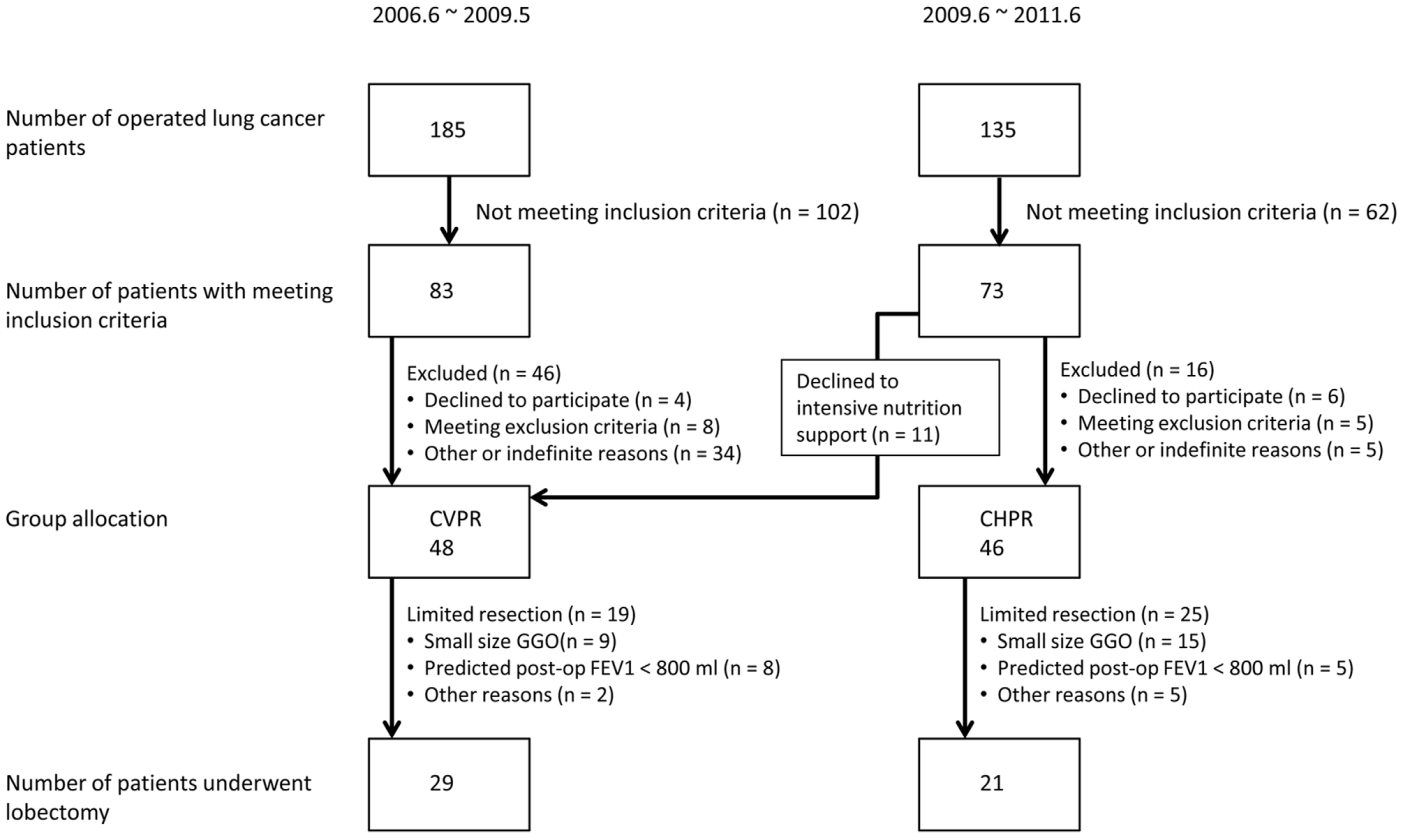
CONSORT diagram for cohort selection.

We started conventional preoperative pulmonary rehabilitation (CVPR) in 2006. CHPR using a multidisciplinary team approach was conducted prospectively from June 2009. Spirometric pulmonary function tests (HI-801, CHEST M.I. Inc., Tokyo) were used to preoperatively assess the baseline severity of the underlying lung conditions. The inclusion criteria for this study were as follows: (1) age >70 years; (2) Vital Capacity (VC)/ideal VC (%VC) <80%; and (3) forced expiratory volume in one second (FEV1)/forced VC (FEV1%) <70%. Patients that met at least one of these criteria were registered in this study from June 2006 to June 2011. Other eligibility requirements included an Eastern Cooperative Oncology Group performance status of 0–1. Exclusion criteria included the presence of an actively treated malignancy or treatment of another malignancy within the past 1 year, presence of a metastatic disease, unstable cardiac disease, and cognitive impairment. All registered patients underwent pulmonary resection after preoperative pulmonary rehabilitation. In CVPR, 48 patients underwent lung resection, and 29 of them underwent standard lobectomy. Nineteen patients in CVPR were treated with limited resection (wedge resection or segmentectomy) mainly because their predicted postoperative FEV1 was less than 800 ml or they had peripheral small-sized ground-glass opacities on computed tomography imaging. On the other hand, 46 patients underwent surgery after CHPR, and 21 of them underwent standard lobectomy for their lung tumors. Twenty-five were treated by limited resection for the abovementioned reasons for the patients in CVPR. Since the amount of resected lung parenchyma itself strongly influenced postoperative outcomes, eligibility for analysis in this study was limited to patients who underwent standard lobectomy for histologically confirmed stage I–IIIA NSCLC. Therefore, the study population consisted of 29 patients in the CVPR group and 21 patients in the CHPR group (Figure 1). Data from the CVPR group were predominantly collected from 2006 to 2009; moreover, 11 patients were registered in the CVPR group after starting CHPR (2009) because they declined the nutritional support (Figure 1).

The demographic data and clinical features of the enrolled patients were collected from the institutional database. Written informed consent was obtained from all enrolled patients, and the protocol of this study was approved by National Hospital Organization Kure Medical Center and Chugoku Cancer Center Institutional Review Board Ethics Committee (Protocol Approval Number; 21–56).

### Protocol for pulmonary rehabilitation

In the CHPR group, the patients were trained to master adequate breathing and coughing techniques, instructed on incentive respiratory exercise, and practiced peripheral muscle exercise training including a cycle ergometer, under the direction of registered physical therapists taking hospital appointments at least twice weekly for 2–5 weeks. The differences in durations between the patients were because of various reasons, such as the choice of date of surgery that was decided by the patients' or for institutional convenience. Registered dieticians assessed the daily diet and directed optimized diet therapy for patients at least twice preoperatively. BCAAs and Hochuekkito^TM^, which is a herbal medicine and composed of 10 nature remedies, were administered daily from the initiation of physical training to discharge after surgery. The daily amount of BCAA supplementation was 6.2 g, which consisted of two packs of supplement (Hepas second^TM^; Clinico Co., Tokyo, Japan or Aminofeel^TM^; Terumo Co., Tokyo, Japan). Registered dieticians chose one of these supplements mainly on the basis of the status of dietary intake, because the amount of total calorie in each of them was different. Multidisciplinary team approach was also accomplished by discussing each patient's conditioning during periodical conferences attended by surgeons, physical therapists, dieticians, and nurses. CVPR consisted only of conventional physical training directed by physical therapists at least once a week preoperatively. Basically, conventional physiotherapy mainly focused on muscle training exercises for improving the activity of daily life. However, there were no apparent differences in the CHPR and CVPR physical therapy programs, except for the minimal required times of hospital appointments. Although the intensity level was usually determined using the Borg scale and the perceived exertion of 13 was the goal intensity level, this level and the quality of physical therapy seemed to have gradually changed over the time period of this study. With the exception of the preoperative pulmonary rehabilitation protocol, standard perioperative management and medications did not changed throughout the study period. Five patients in CHPR were diagnosed with COPD concomitant with lung cancer, and 3 of 5 patients initiated inhalation therapy (Tiotropium bromide hydrate, Boehringer Ingelheim Co., Germany) simultaneously with the CHPR protocol. In the CHPR program, assessment and planning were performed through team conferences and multiple sessions of counseling conducted for each patient. The participants in both groups received uniform, standard postoperative respiratory care and physical therapy until discharge.

### Assessment of preoperative condition

**Figure 2:**
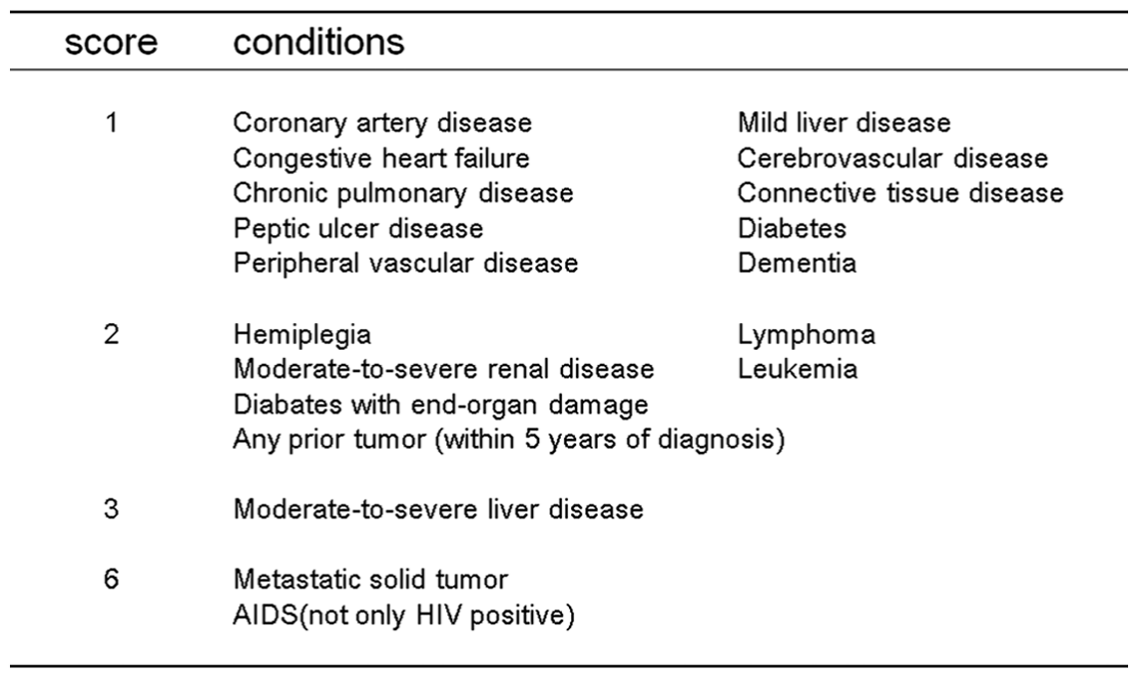
Carlson Comorbidity Index.

**Figure 3:**
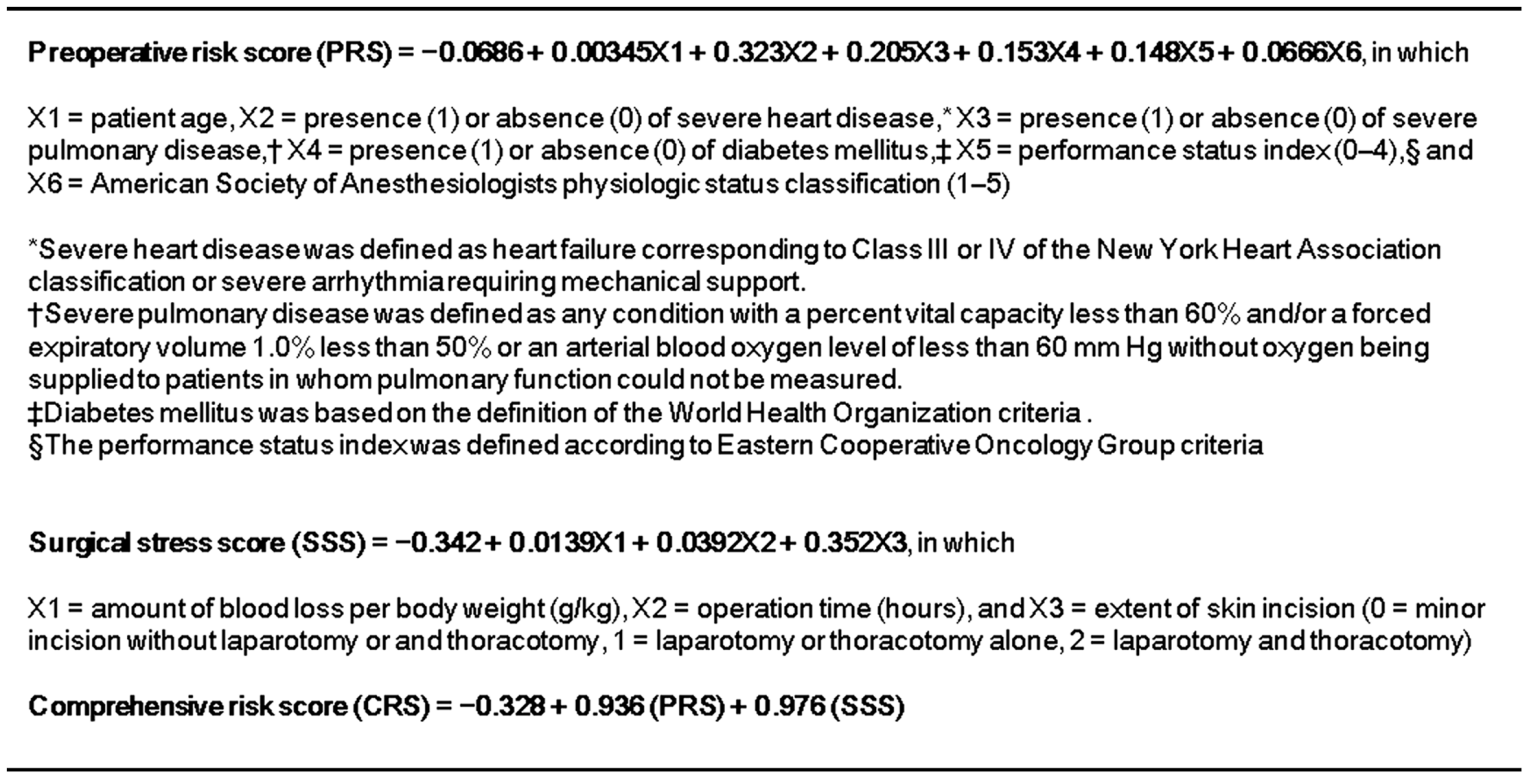
Estimation of Physiologic Ability and Surgical Stress scores.

The preoperative conditions of the patients were assessed by the Charlson Comorbidity Index (CCI) and Estimation of Physiologic Ability and Surgical Stress (E-PASS) scores (Figures 2 and 3) that have been demonstrated to be reliable tools for predicting postoperative outcomes [17], [18]. Because preoperative conditions were the main focus of this study, the preoperative risk score (PRS) defined in the E-PASS was adopted and calculated precisely for all registered patients, even though the E-PASS originally consisted of the PRS, surgical risk score, and comprehensive risk score. Patients with CCI ≥2 or those with PRS >0.3 were designated as being in poor preoperative condition. Comorbidity was assessed by pleural surgeons and physicians, and scores for preoperative conditions were calculated retrospectively in CVPR but prospectively in CHPR. These assessments were blinded for intervention.

### Analyses of postoperative outcome and pulmonary function

In the literature, various rates of postoperative complication have been presented even in patients with equivalent preoperative conditions. These differences appear to have been because of the varying definitions of postoperative complication in each investigation. In this study, the definition of postoperative complication was clearly established (Table 1), and medical records of all registered patients were carefully checked. Practically, relatively common complications graded 2 or higher and relatively infrequent complications graded 3 or higher according to the Common Terminology Criteria for Adverse Events (version 4), which occurred within 30 days of surgery were recorded. Patients with prolonged air leakage for ≥7 days or those who underwent pleurodesis were also recorded. Postoperative complications were assessed though periodical discussions by pleural surgeons and/or the cancer board. To assess the impact of preoperative pulmonary rehabilitation for pulmonary function, spirometry for each patient was conducted at two points: before starting the preoperative rehabilitation program (pre-intervention) and after completion, which was 1 or 2 days before surgery (postintervention before surgery). The transition of the VC and FEV1 values after completion of preoperative pulmonary rehabilitation was analyzed.

**Table 1:** Definition of postoperative complication after lung resection.

Category	Variables	Definition
Pulmonary/Respiration	Pneumonia	≥Grade 2*
	Atelectasis	≥Grade 2*
	Bronchopleural fistula	≥Grade 2*
	Respiratory failure	≥Grade 2*
	Empyema	≥Grade 2*
	Prolonged air leakage	≥7 days, or cases that underwent pleurodesis
	Other complications	≥Grade 3*
Cardiovascular disease	Arrhythmia	≥Grade 2*
	Cardiac failure	≥Grade 2*
	Acute coronary syndrome	≥Grade 2*
	Pulmonary embolism	≥Grade 2*
	Deep vein thrombosis	≥Grade 2*
	Other complication	≥Grade 3*
Other conditions	Cerebral infarction	≥Grade 2*
	Other complication	≥Grade 3*

* According to the Common Terminology Criteria for Adverse Events (version 4).

### Statistical analyses

Comparisons were performed using JMP for Windows (version 9.0) statistical software package (SAS Institute, NC, USA). The results are expressed as means ± standard errors for parameters. Differences in characteristics between CVPR and CHPR were determined using the Mann – Whitney U-test. The transition of pulmonary function after preoperative pulmonary rehabilitation was assessed using a paired t-test. The difference in postoperative complication rate between the two groups was analyzed using Fisher's exact test. Associations among risk factors, postoperative complication, and patient groups were assessed by means of univariate and multivariate logistic regression analyses. Multiple logistic regression analysis was performed using variables found to be p<0.25 by univariate analysis. Differences were considered statistically significant for p<0.05.

## Results

### Effect of CHPR on postoperative outcome

The mean duration and number of sections in each group were 29.1±8.9 days and 8.4±3.4 times in CHPR, 27.9±7.8 days and 6.8±4.5 in CVPR, respectively. The patient characteristics and clinicopathological features are shown in Table 2. No significant differences were observed in all examined factors, including the pathological stages between the CVPR and CHPR patients. The category and grade of postoperative complication is listed in Table 3. Severe complications occurred in 2 patients in the CVPR group. Table 4 shows the effect of CHPR on postoperative outcomes. Complication rate after lobectomy for all patients in the CVPR and CHPR groups was 48.3% and 28.6% (p = 0.2428), respectively. In subgroup analyses of the patients with poor preoperative conditions, patients with CCI ≥2 showed that postoperative complication rate was 68.8% for CVPR and 27.3% for CHPR (p = 0.0341). In patients with PRS >0.3, postoperative complication rate was 57.9% for CVPR and 21.4% for CHPR (p = 0.0362). For all patients, univariate analyses showed that gender, VC, smoking history, surgical approach, and CHPR appeared to be marginal factors for postoperative complication. No independently significant factors were detected by multivariate analyses in this setting (Table 5). Subgroup multivariate analyses showed that CHPR was an independent significant factor for prediction of outcome in the patients with CCI ≥2 (odds ratio, 0.054; 95% confidence interval (CI), 0.001–0.618; p = 0.0157; Table 6). In the patients with PRS >0.3, multivariate analyses showed that CHPR marginally decreased morbidity compared to CVPR (odds ratio, 0.206; 95% CI, 0.031–1.033; p = 0.0549; Table 7).

**Table 2:** Patient characteristics and clinicopathological features.

Factors		CHPR (n = 21)	CVPR (n = 29)	p
Gender	(Female/Male)	9/11	11/18	0.7759
Age		73.7±7.1	72.2±8.1	0.5013
FEV1 (L)		1.96±0.57	1.93±0.55	0.8223
VC (L)		2.77±0.73	2.82±0.74	0.8172
Smoking history	(Current, Ever/Never)	12/9	21/8	0.2224
Approach	(VATS/Open)	16/5	19/10	0.5368
Operative time (min)		255.0±104.0	279.8±64.2	0.3041
Blood loss (mL)		240.0±255.0	269.7±216.3	0.6592
Location	(RU/RM/RL)	7/1/5	15/1/9	0.3043
	(LU/LL)	4/4	1/3	
Histology	(AD/SQ/Others)	14/5/2	17/1/11	0.5088
Pathological stage	(IA/IB)	8/10	7/9	0.5051
	(IIA/IIIA/IV)	4/7/0	1/3/1	
Performance status	(0/1)	13/8	15/14	0.7117
Diabetes mellitus	(+/−)	4/17	6/23	0.8861
COPD	(+/−)	11/10	8/21	0.0872
NYHA classification	(0/I–III)	12/9	22/7	0.2224
Hypertension	(+/−)	10/11	8/21	0.2324
CCI		1.52±1.17	1.76±1.38	0.6484
PRS		0.39±0.13	0.41±0.16	0.6235

CHPR, comprehensive pulmonary rehabilitation; CVPR, conventional pulmonary rehabilitation; FEV1, forced expiratory volume in one second; VC, vital capacity; VATS, video-assisted thoracic surgery; RU, right upper lobe; RM, right middle lobe; RL, right lower lobe; LU, left upper lobe; LL, left lower lobe; AD, adenocarcinoma, SQ; squamous cell carcinoma; NYHA, New York Heart Association;; CCI, Charlson Comorbidity Index; PRS, preoperative risk score in Estimation of Physiologic Ability and Surgical Stress.

**Table 3:** Category and grade of postoperative complications.

Complication		Protocol	
Category	Grade	CHPR (n = 21)	CVPR (n = 29)
Pulmonary/Respiration	2–3	5	10
	4–5	0	2
Cardiovascular disease	2–3	1	0
	4–5	0	0
Others	2–3	0	2
	4–5	0	0

CHPR, comprehensive pulmonary rehabilitation; CVPR, conventional pulmonary rehabilitation.

**Table 4:** Postoperative complication rate in all patients and subgroup analyses.

Group	Protocol	No.	Complication (%)	p
Total	CHPR	21	28.6	0.2428
	CVPR	29	48.3	
CCI ≥2	CHPR	11	27.3	0.0341
	CVPR	16	68.8	
PRS >3	CHPR	14	21.4	0.0362
	CVPR	19	57.9	

CCI, Charlson Comorbidity Index; PRS, preoperative risk score in Estimation of Physiologic Ability and Surgical Stress; CHPR, comprehensive preoperative pulmonary rehabilitation; CVPR, conventional preoperative pulmonary rehabilitation.

**Table 5:** Logistic regression analyses of risk factors for postoperative complication in all patients (n = 50).

Factors		Odds ratio	95%CI	p
Univariate analyses				
Gender	(Female/Male)	0.499	0.141–1.579	0.2349
Age		0.987	0.982–1.065	0.7322
FEV1 (L)		0.984	0.341–2.790	0.9756
VC (L)		1.785	0.808–4.220	0.1537
Smoking history	(Current, Ever/Never)	4.333	1.147–22.447	0.0297
Approach	(VATS/Open)	0.306	0.083–1.051	0.0600
Operative time (min)		1.002	0.995–1.010	0.5186
Blood loss (mL)		1.001	0.997–1.004	0.4043
Protocol	(CHPR/CVPR)	0.427	0.123–1.377	0.1567
Multivariate analyses				
Gender	(Female/Male)	3.347	0.267–88.573	0.3596
VC (L)		1.660	0.573–5.518	0.3519
Smoking history	(Current, Ever/Never)	5.935	0.408–172.436	0.1964
Approach	(VATS/Open)	0.464	0.109–1.896	0.2833
Protocol	(CHPR/CVPR)	0.563	0.145–2.062	0.3862

FEV1, forced expiratory volume in one second; VC, vital capacity; VATS, video-assisted thoracic surgery; CI, confidence interval; CHPR, comprehensive preoperative pulmonary rehabilitation; CVPR, conventional preoperative pulmonary rehabilitation.

**Table 6:** Logistic regression analyses of risk factors for postoperative complication in patients with Charlson Comorbidity Index (CCI) ≥2 (n = 27).

Factors		Odds ratio	95%CI	p
Univariate analyses				
Gender	(Female/Male)	0.476	0.096–2.194	0.3423
Age		0.408	0.029–4.879	0.4798
FEV1 (L)		0.749	0.035–15.302	0.8475
VC (L)		1.903	0.672–6.371	0.2307
Smoking history	(Current, Ever/Never)	4.279	0.852–26.267	0.0782
Approach	(VATS/Open)	NA	NA–0.271	0.0021
Operative time (min)		1.004	0.994–1.016	0.4308
Blood loss (mL)		1.004	0.999–1.012	0.1107
Protocol	(CHPR/CVPR)	0.171	0.027–0.859	0.0315
Multivariate analyses				
VC (L)		2.254	0.320–28.432	0.4246
Smoking history	(Current, Ever/Never)	1.039	0.063–17.133	0.9778
Approach	(VATS/Open)	NA	NA–0.371	0.0134
Blood loss (mL)		0.097	0.983–1.007	0.4998
Protocol	(CHPR/CVPR)	0.054	0.001–0.618	0.0157

FEV1, forced expiratory volume in one second; VC, vital capacity; VATS, video-assisted thoracic surgery; CI, confidence interval; CHPR, comprehensive preoperative pulmonary rehabilitation; CVPR, conventional preoperative pulmonary rehabilitation.

**Table 7:** Logistic regression analyses of risk factors for postoperative complication in patients with preoperative risk score in Estimation of Physiologic Ability and Surgical Stress (PRS) >0.3 (n = 33).

Factors		Odds ratio	95%CI	p
Univariate analyses				
Gender	(Female/Male)	0.550	0.116–2.335	0.4214
Age		0.945	0.831–1.061	0.3387
FEV1 (L)		0.606	0.138–2.364	0.4736
VC (L)		1.535	0.568–4.505	0.3999
Smoking history	(Current, Ever/Never)	3.500	0.678–26.865	0.1397
Approach	(VATS/Open)	0.200	0.039–0.875	0.0323
Operative time (min)		1.002	0.993–1.011	0.6452
Blood loss (mL)		1.001	0.997–1.004	0.8271
Protocol	(CHPR/CVPR)	0.198	0.036–0.878	0.0324
Multivariate analyses				
Smoking history	(Current, Ever/Never)	2.076	0.052–3.480	0.4709
Approach	(VATS/Open)	0.238	0.038–1.221	0.0860
Protocol	(CHPR/CVPR)	0.206	0.031–1.033	0.0549

FEV1, forced expiratory volume in one second; VC, vital capacity; VATS, video-assisted thoracic surgery; CI, confidence interval; CHPR, comprehensive preoperative pulmonary rehabilitation; CVPR, conventional preoperative pulmonary rehabilitation.

### Effect of CHPR on pulmonary function

**Figure 4:**
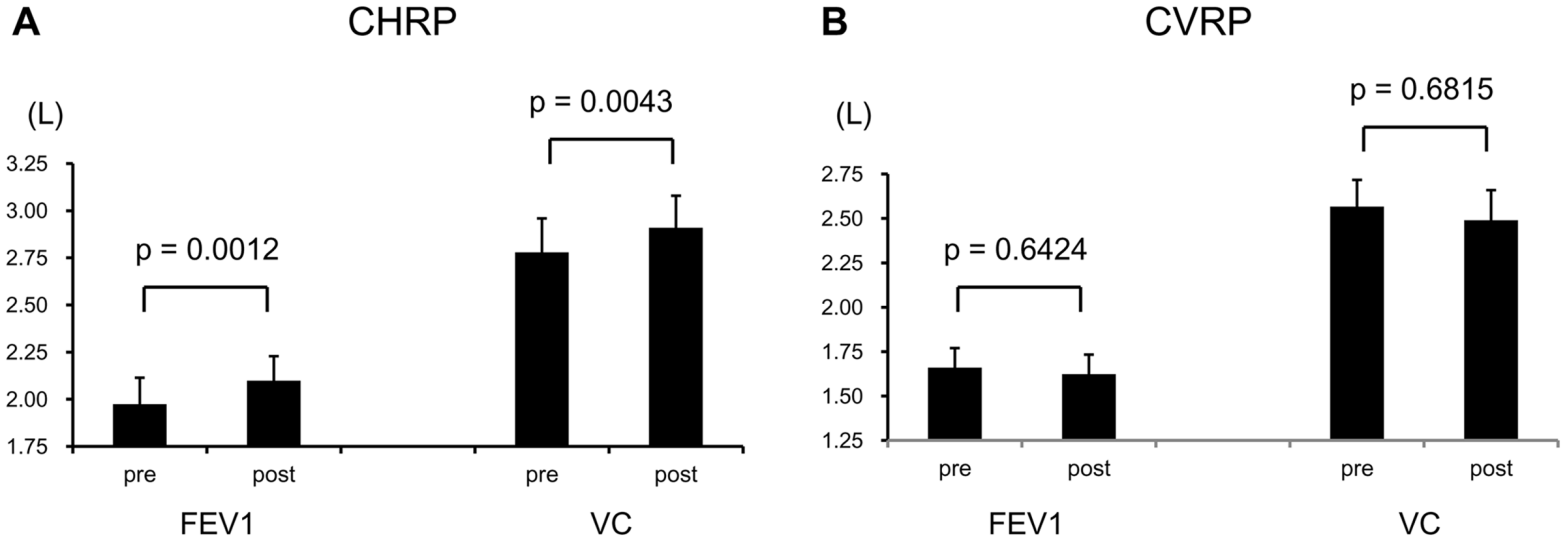
Transitions of pulmonary function after CHPR (A) and CVPR (B). A statistically significant beneficial effect was observed for CHPR. CHPR, comprehensive pulmonary rehabilitation; CVPR, conventional pulmonary rehabilitation; pre, pre-intervention; post, postintervention before surgery; VC, vital capacity; FEV1.0, forced expiratory volume in one second.

A statistically significant beneficial effect on pulmonary function (VC and FEV1) was observed in the CHPR group (p = 0.0043 and p = 0.0012, respectively); however, the transition of those in CVPR was not statistically significant (Figure 4A, B).

## Discussion

The impact of preoperative pulmonary rehabilitation on lung cancer patients planning to undergo lung resection needs to be more clearly investigated, with special emphasis on postoperative complication [19]. Improved perioperative management including physical therapy has contributed to decreasing rates of complication and mortality after lung resection over the past decades [20], [21]; however, comprehensive management strategies for patients are still important issues that require further investigation. To establish an enforceable beneficial program of relatively short-term preoperative pulmonary rehabilitation for outpatients, we developed the CHPR protocol, which consists of physical exercise and intensive nutritional support with BCAAs and herbal medication, conducted using a multidisciplinary team approach. In this study, we found no statistically significant differences in overall postoperative complication rate between CVPR and CHPR, probably because of the relatively small number of registered patients. However, a tendency towards beneficial effects from CHPR on reducing risks was speculated, and preoperative conditions may influence outcome. Additionally, the fact that CCI and PRS in the CVPR group were worse than those in the CHPR group, may have resulted in higher postoperative complication rate in CVPR compared with CHPR. Therefore, subgroup analyses according to the CCI and PRS risk scores were performed. These subgroup analyses showed that CHPR was beneficial in decreasing postoperative complication rate in patients with poor preoperative conditions. Although our patients showed increased values in VC and FEV1 after CHPR compared with CVPR, it is unclear whether these findings of slightly increased lung function could have contributed to the postoperative outcome of the disease. In addition, we conducted CHPR though the multidisciplinary team-based approach to obtain intensive preoperative care. It may also provide beneficial effect on patients as described in literatures [32], [33]. Moreover, it is difficult to know which interventions in CHPR had the greatest influence on the outcome, and the effect of each intervention should have analyzed; however, we considered that each intervention alone did not seem to have a significant potential to improve the post interventional outcome. Therefore, we started the CHPR program on the basis of the idea that comprehensive and a multidisciplinary team-based approach should be considered for the development of clinically available and a more effective preoperative pulmonary rehabilitation program.

The most frequently advocated obstacle in scheduling preoperative pulmonary rehabilitation for lung cancer patients is the necessity to perform surgical treatment without delay, taking into consideration the potential for malignancies. Recent literature demonstrated that a 10-session preoperative pulmonary rehabilitation may improve postoperative lung re-expansion and decrease the length of hospital stay; however, a 4-week preoperative program proved to be very difficult to apply [22]. Cesario and colleagues found that in an inpatient program for patients selected at the surgeon's discretion, significant improvements were observed in forced VC, 6-min walking distance (6 MWD), and partial oxygen pressure in arterial blood after five daily sessions of 3 h each [23]. Jones and colleagues demonstrated improvement of the volume per time oxygen maximum and 6 MWD after five sessions per week for 3 weeks [24]. Bobbio and colleagues reported an improvement in exercise capacity despite an absence of changes in the resting FEV1 after 90-min sessions for 5 days over 4 weeks [25]. Weiner and colleagues performed a prospective randomized controlled trial that involved training for 1 h per day for 2 weeks and reported that the intervention group had better predicted postoperative FEV1 than the control group 3 months after surgery [26]. These studies indicated that physical therapy based preoperative pulmonary rehabilitation improved exercise capacity even in the short term, although the data were inconsistent in the different study designs and protocols. Although such literatures demonstrated that preoperative physical therapy provided beneficial effect, development of an advanced and effective multidisciplinary protocol for a short-term preoperative pulmonary rehabilitation is still an important issue, especially for patients with poor preoperative condition.

Nutritional support for improving exercise performance has been recognized as an important intervention, especially for physically deconditioned individuals including those with poor pulmonary function [27]. The majority of lung cancer patients have long smoking histories; therefore, there is a significant prevalence of COPD in these patients, approximately 73% in men and 53% in women [6]. A growing body of evidence suggests that COPD patients generally show lower plasma BCAA concentrations than control patients [28], suggesting that COPD, which is characterized as a chronic wasting disease with a high metabolic rate, may benefit from BCAA administration. Clinical data are available, which have shown the beneficial effect of BCAA supplementation on patients with chronic heart failure [29], surgery [30], and diabetes [31]; however, the potential role of BCAA supplementation in preoperative pulmonary rehabilitation for lung cancer patients remains to be elucidated. Recently we began to calculate 6MWD and the transition of it, which assessed in a limited number of patients with CHPR, showed considerable improvement (data not shown). It could be presumed that BCAA supplementation probably influenced the results of our study because the CHPR group may have tolerated the intensive physiotherapy better than the CVPR group. To the best of our knowledge, this study is the first assessment of the clinical benefit of BCAA supplementation in preoperative pulmonary rehabilitation program. Furthermore, to accomplish intensive nutritional support, herbal medications were also administered in this study. Interestingly, the effect of such medication on maintenance or improvement of appetite and in terms of quality of life has been evaluated among patients with COPD [16]. Recently, we started analyzing the transition of nutritional condition in CHPR. Although a sample size was limited, most patients showed a decreased body weight but an increased muscle volume using the body fluid measurement (data not shown). In addition, we started analyzing the serum level of rapid turnover proteins and several amino acids. Although the data are still insufficient to be demonstrated because of the limited sample size, the level of retinol binding protein increased in 7 of 10 patients after CHPR.

There were several limitations of this study that should be noted when assessing the results. First of all, although the CHPR protocol was adopted prospectively, most of the CVPR data were collected retrospectively. The number of patients included in each cohort was relatively small, and the assignment of the patients to the two groups was not random. It is important to note that data of CVPR and CHPR were collected at predominantly different time periods, although 11 patients in CVPR were registered after starting the CHPR program. In addition, other various factors including exercise capacity, QOL, and nutritional variants need to be evaluated for elucidating potential benefit of the CHPR protocol. Moreover, this study was based on the data of patients at a single institution. Taken together, we demonstrated the clinical benefit of the CHPR protocol with short-term preoperative pulmonary rehabilitation in this study; however, prospective randomized studies should be conducted before such treatment can be recommended in routine clinical practice. At present, a multicenter, prospective feasibility study is being conducted by the study group of national hospital organizations in Japan for evaluating the clinical effect of CHPR via a multidisciplinary team approach.
